# Purpura Hæmorrhagica in a Dog

**Published:** 1898-05

**Authors:** Wilfred Lellman

**Affiliations:** Professor of Materia Medica and Therapeutics and Parasites and Parasitic Diseases, New York College of Veterinary Surgeons, New York City


### REPORTS OF CASES.

### MORBUS MACULOSUS (PURPURA HÆMORRHAGICA) IN A DOG.

By Wilfred Lellman, D.V.M.,
PROFESSOR OF MATERIA MEDICA AND THERAPEUTICS AND PARASITES AND PARASITIC DISEASES, NEW YORK COLLEGE OF VETERINARY SURGEONS, NEW YORK CITY.

On April 19th of last year I was requested to examine a dog which had been sick for about two weeks. According to the owner's history, the animal had had very poor appetite, and had shown considerable depression. Within the last three days a remarkable swelling of the abdomen had appeared.

A thorough clinical examination was made, the result being as follows: Male mastiff, about six years old, in poor state of nutrition; hair erect and lustreless. The conjunctival membrane highly anæmic; pulse small and wiry, irregular, beating about 100 times per minute. The heart-beat is weak, still it can be felt, as the patient was considerably emaciated. Percussion showed enlarged dulness of the heart-region. While auscultating the heart I heard two by-sounds, one during the systole, the second during the diastole. The first one I considered due to an insufficiency of the mitralis, the second one to the pressure of pericardiac liquid. The rectal temperature is 100° F., the surface of the body appeared to have subnormal temperature; the nose is dry. The respiration appeared dyspnœic, thirty-five times per minute. Percussion of the thorax shows several regions of dulness. Auscultation of the lungs proves sharp vesicular and bronchial bruits. When examining the mouth I detected numerous hemorrhages and ulcerations of the gums and the buccal mucous membrane. A very offensive odor diffuses from the mouth. The tongue appeared dry, and would not thrust off epithelia. The abdomen shows an immense swelling. Percussion shows remarkable dulness. When palpating the abdomen I found a considerable quantity of liquid within, also enlargement of the liver and spleen. The defecation is retarded, the feces being of hard consistency, of yellowish-grayish color, covered with mucus, which shows small streaks of blood. The animal has no appetite, but increased thirst. The urine is of yellowish-red color, specific gravity 1060. By means of Esbach solution I detected 1 per cent. albumin; sugar is not present. A microscopical examination shows red blood-corpuscles, white blood-corpuscles, epithelial casts, which are partially covered with red blood-corpuscles; epithelial cells of the bladder are also found.

The psyche of the patient shows considerable depression. The posterior extremities show a slight œdematous swelling. The blood was examined by means of the Thoma-Zeiss hæmacytometer; nothing particular, except from anæmia was found; the properties of the red blood-corpuscles and the white ones are scarcely altered. In order to make the diagnosis sure I made a test puncture of the abdominal cavity. The liquid proved to be a bloody exudation. As for the differential diagnosis, morbus maculosus and scorbutus could only be taken into consideration. At present it is hard, from the clinical standpoint, to draw a distinct line between these diseases, as the etiology of these hæmorrhagic diseases is quite obscure. Under the circumstances, it must be left to the judgment of the veterinarian whether he has to deal with morbus maculosus or scorbutus. Though human pathology considers ulcerations of the gums as something characteristic of scorbutus, we have to admit that they can also be met with in morbus maculosus. Generally, I think, we are right when considering scorbutus of not such acute and grave character as morbus maculosus. Concerning the etiology of scorbutus, we presume that it is due to irrational and poor food. This, however, was to be excluded in this case, as I informed myself thoroughly about the way in which the dog was nourished. The possibility of being poisoned would also be excluded, according to the positive assurance of the owner.

Prognosis, of course, was very bad. The animal lived for four days longer, then all at once acquired a severe epistaxis. Exitus vitalis under the symptoms of hemorrhage.

An autopsy was held immediately after death, the result being as follows: Abdominal cavity contains two and one-half grammes of bloody fluid, hemorrhages in the submucosa of the stomach and intestinal tract, numerous ecchymoses under the peritoneum, hemorrhages within the liver, kidneys, spleen; liver immensely enlarged, spleen also quite considerably. The pericardium contains about one-half pint of bloody liquid. As further pathological alterations, I mention endocardial hemorrhages, insufficiency of the mitralis. The lungs showed hemorrhagic infarcts and severe chronic bronchitis. Hemorrhages within the cutis, subcutaneous tissue, muscles; articulation could not be found.

