# Correction

**Published:** 1898-06

**Authors:** 


CORRECTION.

In the May number, on page 327, line 24, instead of " would not thrust off epithelia," read " covered with thrust-off epithelia ;" line 32, instead of " 1 per cent. albumin, read " 1 per mille albumin."

On page 328, line 11, instead of " hæmorrhagic diseases," read hæmorrhagic diatheses ;" line 26, instead of " *exitus vitalis,*" read " *exitus letalis.*"

